# Simultaneous resonant x-ray diffraction measurement of polarization inversion and lattice strain in polycrystalline ferroelectrics

**DOI:** 10.1038/srep20829

**Published:** 2016-02-11

**Authors:** S. Gorfman, H. Simons, T. Iamsasri, S. Prasertpalichat, D. P. Cann, H. Choe, U. Pietsch, Y. Watier, J. L. Jones

**Affiliations:** 1 Department of Physics, University of Siegen, Siegen, 57072, Germany; 2 European Synchrotron Radiation Facility, Grenoble, 38043, France; 3 Department of Physics, Technical University of Denmark, Lyngby kgs. 2800, Denmark; 4 Department of Materials Science and Engineering, North Carolina State University, Raleigh, NC, 27695, USA; 5 Materials Science, School of Mechanical, Industrial, and Manufacturing Engineering, Oregon State University, Corvallis, OR 97331, USA

## Abstract

Structure-property relationships in ferroelectrics extend over several length scales from the individual unit cell to the macroscopic device, and with dynamics spanning a broad temporal domain. Characterizing the multi-scale structural origin of electric field-induced polarization reversal and strain in ferroelectrics is an ongoing challenge that so far has obscured its fundamental behaviour. By utilizing small intensity differences between Friedel pairs due to resonant scattering, we demonstrate a time-resolved X-ray diffraction technique for directly and simultaneously measuring both lattice strain and, for the first time, polarization reversal during *in-situ* electrical perturbation. This technique is demonstrated for BaTiO_3_-BiZn_0.5_Ti_0.5_O_3_ (BT-BZT) polycrystalline ferroelectrics, a prototypical lead-free piezoelectric with an ambiguous switching mechanism. This combines the benefits of spectroscopic and diffraction-based measurements into a single and robust technique with time resolution down to the ns scale, opening a new door to *in-situ* structure-property characterization that probes the full extent of the ferroelectric behaviour.

The large dielectric coefficients and strong electro-mechanical coupling of ferroelectric materials derive from the spontaneous polarization of their asymmetric crystalline structure and ability to switch between two or more polarization states under externally applied electric fields. Polycrystalline ferroelectrics offer a cost-effective and versatile route to these functionalities and are the backbone of many sensors, actuators, non-linear optics and power conversion devices^1^. The interconnection of polarization reversal with electromechanical coupling is complex and exists across several length and time scales, due to the combined influences of the intrinsic crystalline structure, the polarization reorientation mechanism(s) and localised effects such as neighbouring grains or defects. Understanding and de-convoluting these factors is key to the optimization and discovery of new materials with enhanced properties. However, existing techniques lack the capability to directly measure polarization-strain coupling across the relevant spatiotemporal regime.

While ferroelectric polarization reversal is essentially an electric field induced structural inversion (180° switching), it may occur through different and sometimes competing mechanisms. One mechanism involves the motion of a domain wall - a planar defect separating two volumetric domains of uniform and antiparallel spontaneous polarizations. Moving 180° domain wall via the application of an electric field changes the volumetric ratios of oppositely polarized domains and hence the net polarization. Another mechanism may involve intermediate steps such as successive 90° domain switching events and/or ferroelastic phase transitions^2^,^3^,^4^. A third mechanism is related to the transformation between ordered and disordered states such as in relaxor ferroelectrics^5^,^6^,^7^,^8^. In the presence of a strong and rapidly applied electric field, these mechanisms may be suppressed in favour of an entirely homogeneous switching^9^. Experimentally de-convoluting these four mechanisms of polarization reversal remains challenging, and the lack of knowledge in this area hinders design and control of preferential polarization reversal routes.

Simultaneously inspecting both the lattice strain and polarization response of polycrystalline ferroelectrics *in-situ* during domain switching would therefore be a major step forward. The independent study of the competing electromechanical coupling mechanisms within the bulk material would then enable validation of molecular to multi-scale models that incorporate the coupled structure-property relationship in this important class of material.

Such a study can be based on high-energy X-ray diffraction; an established technique that can penetrate μm to mm into a material and provide detailed information of how structure influences properties without spurious surface effects. For example, both 90° domain wall motion and lattice strain may be quantified *in-situ* from the intensity and angular changes of Bragg reflections^2^,^4^,^10^,^11^,^12^,^13^,^14^, while changes to the diffuse X-ray scattering^15^,^16^ enable characterization of the changing order-disorder parameters. Additionally, the displacements of atoms within the unit cell can be probed through structure factor analysis of the variation of Bragg intensities from single crystals under electric fields^17^,^18^,^19^,^20^. Time-resolved X-ray diffraction is also a powerful tool for measuring structural dynamics (e.g.^21^,^22^).

A useful yet largely unexplored potential of X-ray diffraction is its sensitivity to structural inversion arising from the resonant (anomalous) scattering^23^,^24^,^25^. Friedel’s law states that the structure factors of Friedel pair reflections (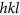 and 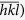 are equivalent, implying that inversion-related structures cannot be differentiated in an X-ray scattering pattern. However, Friedel’s law is broken in the case of resonant X-ray scattering, and intensities of Friedel pair reflections differ slightly. Polarization reversal in ferroelectrics is therefore measurable from these intensity changes.

Despite its potential, the experimental realization of resonant X-ray scattering has been difficult. Observing contrast between Friedel pairs requires highly precise measurements of diffraction intensities, and thus has been primarily applied in single crystals^26^ and epitaxial thin films^27^,^28^. In the case of single crystals, absorption, extinction and multiple scattering often hinder precision, and successful applications of this remarkable technique are rare^26^,^27^,^28^,^29^. Both extinction and multiple scattering effects are absent in the case of powder diffraction because typical powder crystallites sizes are below the critical length of dynamical scattering. However, overlapping 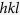 and 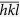 Debye-Scherrer rings means measuring such Friedel pair contrast using powder diffraction has never been considered, despite the fact that the crystallographic structure of their powder crystallites can be actively inverted by an external electric field in the same manner as single crystals. This inversion provides the opportunity to observe the Friedel pair contrast simply by the application of an electric field.

Here we present an example of the use of resonant synchrotron X-ray diffraction to directly and simultaneously measure the dynamic strain and, for the first time, the ionic response of a polycrystalline ferroelectric to cyclic electric fields. We demonstrate this approach using a high-energy (30 keV) X-ray beam on a material in which differentiating between multiple polarization reversal mechanisms remains a significant challenge: tetragonal 0.94·BaTiO_3_-0.06·BiZn_0.5_Ti_0.5_O_3_ (BT-BZT) perovskite-based polycrystalline ferroelectrics^30^. Using structure factor simulations, we then directly correlate the measured changes of the {111} Bragg intensity to the atomic displacements and hence relative polarization changes in the sample. Ultimately, the approach offers significant opportunities for probing the dynamics of intrinsic polarization using resonant X-ray scattering.

## Results

**Figure 1:**
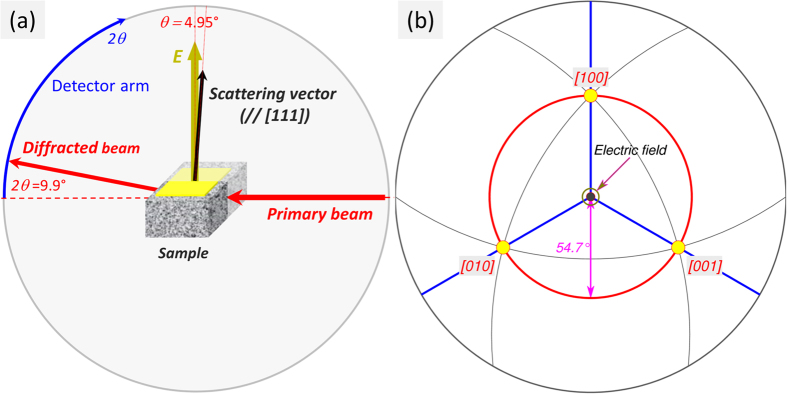
Schematic orientation diagram of the electric field, scattering vector and polarization directions. (**a**) Diffraction geometry for {111} -oriented tetragonal grains. The electric field, ***E***, is applied at the *θ* = *4.95°* to the [111] direction of the scattering vector. (**b**) Stereographic projection (viewed along [111]), showing three polarization directions ([*100*], [*010*] and [*001*] - yellow filled circles) in the {*111*} - tetragonal powder grains. The angle between any polarization direction and the scattering vector is 54.7°. The almost equal inclinations of these three polarization directions to the electric field imply that electric field-induced exchange between them (*i.e.* 90° domain wall motion) is not expected.

In this technique, X-rays are diffracted by the individual tetragonal crystallites embedded in the polycrystalline matrix whose {111} Miller planes are at the Bragg angle (q) to the primary beam, and thus almost perpendicular to the electric field (Fig. 1a). Each tetragonal crystallite includes three types of 90° domains, whose spontaneous polarization vectors are aligned to the [100], [010], [001] directions. Figure 1b shows that these directions are almost equally inclined to the electric field (at the ‘magic’ angle: 
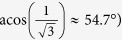
, meaning that exchange between these domains (90° domain wall motion) is not expected. Therefore, in the absence of elastic interactions from neighbouring material, only 180° domain wall motion is expected in these crystallites. Further description of the experimental methodology is provided in Methods.

**Figure 2:**
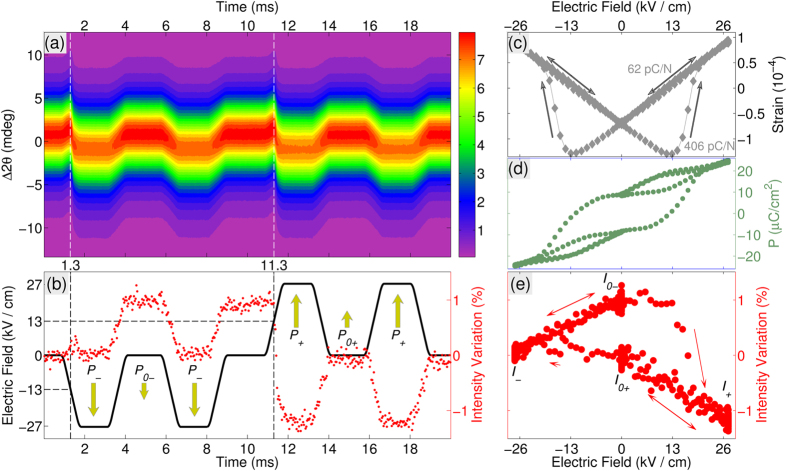
Combined behaviour of the {111} diffraction profile under applied electric field. (**a**) False-colour map of the diffraction intensity (arbitrary units) plotted as a function of time (horizontal axis) and scattering angle (vertical axis). The vertical dashed lines mark the switching times, identified as maximums of the average peak position/minimums of the lattice spacing; (**b**) the time-dependence of the applied voltage (+/−27 kV/cm) (black line) and the variation of the integrated intensity (red dots); The switching times (1.3 ms and 11.3 ms) and the corresponding switching fields (−13 kV/cm and +13 kV/cm) are marked by the dashed lines. The resulting polarization states are marked by the arrows. (**c**) voltage dependence of the strain (the relative change of the a_111_ lattice spacing), showing a typical butterfly hysteresis loop - the intrinsic piezoelectric constants, corresponding to two different parts of the loop are explicitly marked. The local minima of the loops define the magnitude of the coercive field (13 kV/cm). (**d**) the electric-field dependence of the macroscopic polarization (P-E loop). (**e**) the electric field dependence of the integrated intensity, passing four of the above polarization states.

Figure 2a shows the time-resolved {111} powder diffraction profile during the application of a 50 Hz cyclic electric field comprising two negative and two positive pulses. The time-dependence of the applied electric field is shown by the thick solid line in the Fig. 2b: in such a double-pulse waveform, the first pulse probes the switching response (polarization reversal), while the second pulse probes the dielectric response (polarization extension) as schematically shown by the vertical arrows. The systematic time-dependent shift of the {111} profiles is directly obvious from the Fig. 1a. The {111} profile remains single throughout the electric field cycle: this suggests that the field-free tetragonal symmetry of the material is conserved under electric field. The position (the centre of mass) of a powder diffraction peak is solely defined by the single {111} lattice spacing: 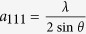. The shift of the peak 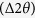 means the change of this spacing 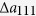 and development of the corresponding strain component: 
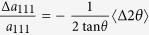
.

The ‘butterfly’ shape of the strain-field loop, 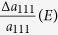, (Fig. 1c) shows that the behaviour of the material is classically ferro- and piezoelectric. Furthermore this loop enables direct determination of the coercive field for polarization reversal (13 kV/cm, corresponds to the minima of the ‘butterfly’ loop) and the piezoelectric constant as 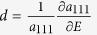
during the linear strain-field intervals. In the interval immediately following the polarization reversal, the piezoelectric response exhibits an increase of 6.5 times.

Electric field dependence of the macroscopic polarization (*P-E* loop) (Fig. 2d) confirms the coercive field of 13 kV/cm. However, the *P-E* loop describes the entire volume of the sample and, therefore, includes the contribution of the 90° domain wall motion. On the contrary, {111} powder diffraction selects those crystalline grains, in which 90° domain wall motion is not expected (Fig. 1). This important difference accounts for the presence of unipolar hysteresis loops in the *P-E* data (Fig. 2d) and their absence in the strain-field data (Fig. 2c).

The ionic polarization response is represented by the integrated intensity of the {111} profile (Fig. 2b,e) through the change of the structure factors/positions of atoms in the unit cell. Significantly, evidence of polarization reversal and the resulting breaking of Friedel’s law due to resonant scattering exist in the field-induced integrated intensity changes. Two pairs of mutually reversed characteristic polarization states 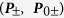 can be inferred from the resonant intensity changes: 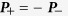
and 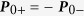 represent the 27 kV/cm- field and zero-field (remnant) states, respectively. The ~1% intensity contrasts, 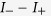 and 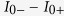 between “−” and “+” states significantly exceeds the 0.2% uncertainty estimated from the Poisson statistics. The integrated intensity changes as a function of applied electric field is highlighted in Fig. 2d.

**Figure 3:**
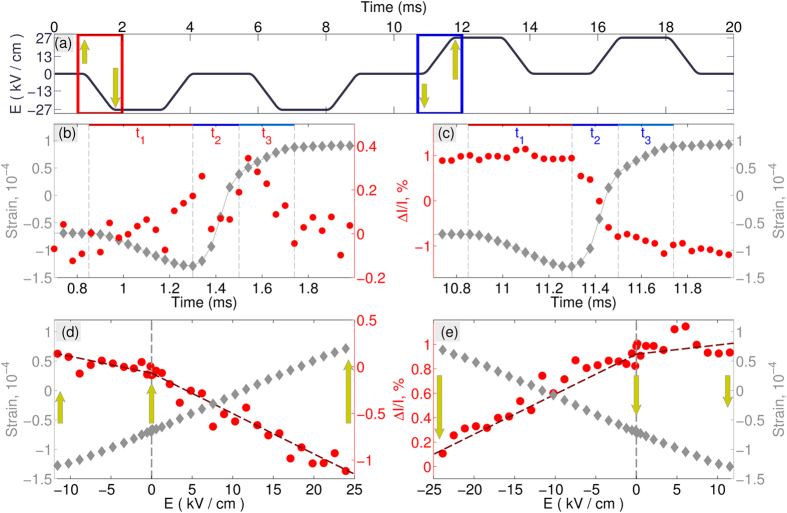
The details of the time and field dependence of the strain and Bragg intensity response. (**a**) Time-dependence of applied electric field with two highlighted intervals of interest, featuring the negative and the positive switching pulse. The change of the polarization vector during these intervals is schematically shown by the vertical arrows. (**b,c**) Time-dependence of the strain and the integrated intensity, during the negative (**b**) and the positive (**c**) switching pulses, highlighted in (**a**). Three time intervals, describing the course of the polarization reversal (t_1_, t_2_, t_3_) are shown: the intervals t_1_ and t_3_ corresponds to the pre-reversal and after-reversal stages correspondingly; (**d,e**) demonstrate the non-switching field dependence of the strain and intensity, highlighting the different responses of intensity during application of fields, parallel and anti-parallel to the polarization direction. The polarizations are schematically shown by the vertical arrows.

This simultaneous measurement of the strain and ionic polarization gives new insight into the dynamic response of the material under the field application. These combined responses during the positive and negative switching events are detailed in Fig. 3(a–c). The time-dependence of the polarization reversal process (Fig. 3b,c) exists in three distinct stages: *t*_*1*_ - where the weak electric field opposes the initial polarization direction, *t*_*2*_ – where the field exceeds the coercive field and switching is evidenced by the sharp change to the piezoelectric response, and *t*_*3*_ – where switching has completed and the electric field is parallel to the polarization direction.

The electric field-dependent lattice and intensity response provides the further insight into the dynamics of atomic positions (Fig. 3d,e) in the non-switching regime. While lattice strain varies linearly with field magnitude, the intensity response tells a different story: greater atomic displacements occur when electric field is oriented along the polarization direction than when opposing it. The same asymmetric response to an electric field can be seen in the *P-E* loop (Fig. 2d).

This has important implications in terms of the atomic displacement response to an electric field and, crucially, enables the development of a structural model of the polarization reversal process.

## Discussion

### Structural modelling of the polarization reversal

The observed intensity changes can be quantitatively related to the atomic displacements through the structure factor. According to kinematical diffraction theory, the integrated intensity of diffraction by a single domain crystallite is proportional to the squared structure amplitude, 
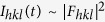
:


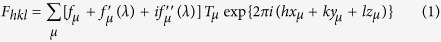


where 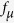 and 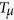 are atomic scattering and Debye-Waller factors correspondingly, 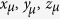 are the fractional positions of the 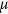
's atom in a unit cell, 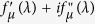 are the wavelength dependent resonant scattering corrections. The intensity 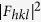 is sensitive to structural inversion only when the atoms with non-zero 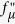 are present. For the BT-BZT at a wavelength of 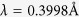, these atoms (and their 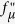 values) are Ba(0.82), Bi(4.19), Zn(0.50) and Ti(0.14)^31^.

**Figure 4:**
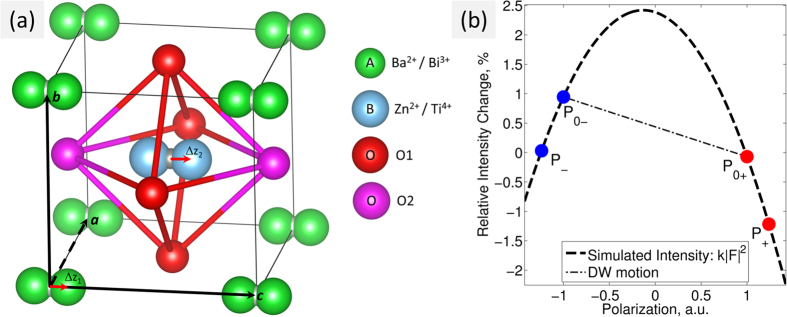
Schematic view of the tetragonal (*P4mm* space group) perovskite unit cell and the simulated 1 1 1 Bragg intensity change due to displaced atomic positions. (**a**) The tetragonal perovskite unit cell, described by the atoms, distributed at four symmetry independent crystallographic sites. The polarization is modelled by the off-centre displacements of *A*-positions by ∆z_1_ and B-positions by ∆z_2_. The oxygen octahedra cages are kept undistorted. (**b**) Simulated intensity change (the bold dash line) shows the polarization dependence of the structure factor 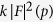, where 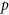 scales the off-centre displacements of atoms on the A and B sites, so that 
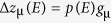
. The coloured circles depict the observed intensities during four polarization states.

Figure 4a shows the unit cell of the ABO_3_ tetragonal perovskite-type structure with the *A* sites, occupied by Ba^2+^/Bi^3+^, and the *B* sites, occupied by Zn^+2^/Ti^4+^ ions. We modelled the structural response to the electric field by assuming a fixed oxygen framework and shifting the A (μ = 1) and B (μ = 2) cations along the polar axis relative to their ideal perovskite 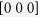 and 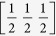 positions respectively. Assuming that these shifts 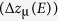, belong to a common optical phonon mode, we scale them by an electric field dependent normalized polarization factor 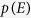 so that 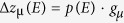, where 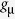 are the model parameters describing spontaneous polarization due to atomic displacements. The normalized polarization factor, 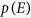, can be defined in such a way that 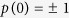 and 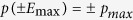 where 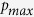 is another model parameter and 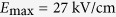. The normalized polarization is directly connected with the net macroscopic polarization, 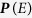, of a single domain according to:


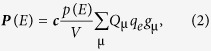


where *V* is the volume of the unit cell and 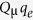 are atomic charge of the *μ*'s atom, 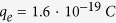 is an elementary charge, ***c*** is the corresponding crystallographic basis vector. Because the atomic charges are constant, the dynamics of the net polarization, 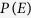, must be identical to the dynamics of the normalized polarization 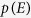.

We first analyse the observed diffraction intensities, 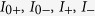 during the four stable states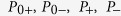 as marked in the Fig. 2(b). The shifts of the A and B cations during these four states can be described in terms of three parameters 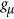 *(μ=A, B)* and 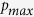 as 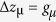, 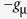,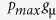, 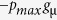 respectively. We then used equation (1) to find the values of these parameters as 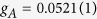, 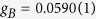, 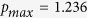 by minimising the difference between the observed intensities (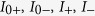,) and their corresponding squared structure amplitudes 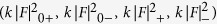, where *k* is the scale factor. This corresponds to the zero-field off-centre displacements of *A* and *B* sub-lattices by ~0.2 Å and their extension of 23.6% as the electric field amplitude increased from 0 to 27 kV/cm. It is then possible to estimate the net remnant macroscopic polarization of a single domain according to (2) as


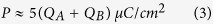


where 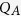 and 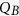 are average atomic charges (in the units of 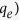, corresponding to the atoms at the A and B positions. Although 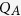 and 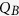 are unknown, assuming that 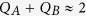, gives a close match to the macroscopic polarization, displayed in the Fig. 2d.

We must stress that these off-centre atomic shifts could not be determined without resonant scattering effects, and the squared structure amplitudes differed between positive and negative polarization states.

Understanding the relationship between the structural polarization and the intensity changes can reveal fundamental mechanism of the switching process. The dashed line in Fig. 4b shows the expected change in intensity, 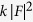 with variation to the normalized polarization 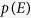 resulting from the cation displacements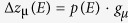. The filled circles pinpoint the experimentally-measured polarization states, 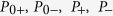. In the case of homogenous structural switching (i.e. passing of the atomic displacements 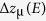 through zero) between 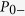 and 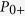, the intensity response would follow the dashed parabolic arc. On the other hand, polarization reversal through 180° domain wall motion (*i.e.* changes to the volumetric ratio of the 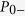 and 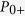, domains) would follow the straight dotted line. In principle, it is therefore possible to use this method to determine the switching mechanism by measuring the intensity changes during the switching process.

### Polarization dynamics

**Figure 5:**
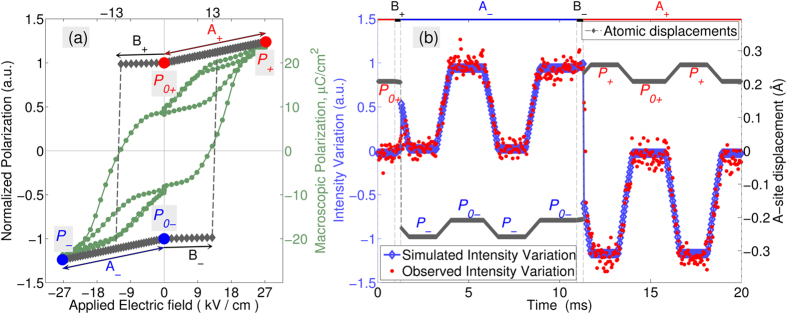
Time and field dependence of the intrinsic polarization/atomic displacements and corresponding 1 1 1 diffraction intensity variation. (**a**) Electric-field dependence of the normalized ‘structural’ polarization (diamonds), along with the macroscopically measured polarization (circles). (**b**) Simulated (blue) and observed (red) intensity variation. The grey curve shows the time-dependence of the displacement of atoms on their A-sites.

We can infer further details about the dynamics of the intrinsic polarization, 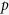, from the time-dependence of the diffracted intensity. The dynamic response is described here in four regimes: A_+_ and A_-_, during which the polarization is parallel to the field (i.e. purely dielectric response without switching) and B_+_ and B_-_, where the intrinsic polarization is antiparallel to the field (and less than *E*_*c*_). These regimes are illustrated in the context of the polarization hysteresis loop 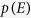, which represents the change of atomic positions during the entire high-voltage cycle (Fig. 5a), i.e. between the reference 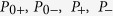 states (marked by circles). Local structural dielectric susceptibilities exist, 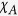 and 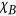 which are associated with these regimes (marked by the arrows) such that 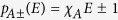, and 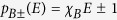, where 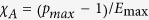, and 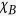 is significantly smaller than 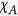 (for the current simulation 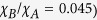. The structural polarization reversal process was then modelled assuming an instantaneous switching of the polarization response from 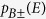 to 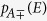. The fact that 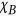 is significantly smaller than 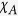 is also supported by the macroscopic P-E loop (included in the Fig. 5a). It is important to note, however, that P-E loop is the combined effect of atomic displacements and 90° domain wall motion and cannot be directly corresponded to the structural *p(E)* loop.

Figure 5b compares the observed (red points) and calculated (blue symbols) intensity changes corresponding to the time and voltage dependence of the intrinsic polarization. The plot shows the remarkable agreement between the model and the experimental intensity data. The main deviation between the simulated and observed intensity occurs during the switching time intervals (i.e. immediately following *B_+/−_*). This however provides potential insight into the switching process. As illustrated in Fig. 4b, the homogeneous structural switching route must be accompanied by a 2.5% intensity increase, which is not observed here. There are two reasons the model makes such an overestimation: 1) either polarization reversal (and corresponding rise of intensity) must occur at time scales less than the 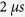 time resolution of the experiment, or 2) polarization reversal does not occur homogeneously and the net normalized polarization, 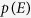, never passes through zero state in the sampled grains. Given the lack of evidence of homogeneous switching in prior works and the fact that previously reported switching processes occur in the μs time scale, we conclude the polarization reversal is likely to follow a 180° domain wall motion mechanism, upon which the volumetric ratios of the states 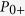 and 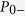 are modified under electric fields.

Resonant X-ray diffraction opens a door to new experiments in a broad class of dielectric materials, and further study could enable determination of polarization inversion mechanisms in the vast majority of ferroelectric materials. Further experiments with better time resolution that sample more reflections would also de-convolute the contribution of homogeneous structural switching mechanisms in 180° domain wall motion. Since ferroelectrics often offer complementary ferroic functionality in multi-ferroic systems, the switching responses to the other perturbing fields (e.g., stress or magnetic field) may also be of interest. While the measurements of a small intensity changes requires high-brilliance X-ray sources, this technique will be routinely possible with the emergence of the new generation of high-intensity X-ray sources (e.g. MAXIV, ESRF upgrade). Another result is the analysis framework can be applied to other techniques such as single crystals, 3D X-ray microscopy and imaging.

### Conclusion

We used the resonant X-ray diffraction to simultaneously measure the structural distortions underpinning intrinsic strain, spontaneous polarization and dielectric response in BT-BZT ferroelectric ceramics. For the first time, we have shown it is possible to measure the Friedel’s pair contrast by high-energy X-rays using powder diffraction. Because the 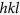 and 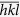 powder rings exactly overlap with one another, the conditions at which the violation of Friedel’s law can be observed using a powder diffraction are very rare. These conditions can only be realized if: 1) a structure of individual powder grains can be actively inverted during the measurement and 2) the intensity of powder diffraction patterns is high enough to detect this small difference. Our findings show that the investigation of the entire, structurally-derived functionality of ferroelectric powders and ceramics is now readily achievable.

We showed a homogeneous spontaneous polarization in BT-BZT can be accounted for by the average off-centred displacement of A and B cations by ~0.2 Å along the polar axis, relative to the oxygen octahedra. In doing so, we demonstrated that polarization reversal in this material must be completed during a time interval that is either shorter than 2 μs (time-resolution of the experiment) or, if not, likely to occur through 180° domain wall motion.

## Methods

A ceramic sample of composition 0.94·BaTiO_3_-0.06·BiZn_0.5_Ti_0.5_O_3_ (BT-BZT) were prepared via conventional solid state synthesis^30^. Its dimensions were 1.10 mm (between the electrodes) × 1.23 mm (along the X-ray beam) × 8.6 mm (width). The electrodes were painted by a silver paste; the sample was not poled before the experiment. *In-situ* powder diffraction measurements were carried out at beamline ID22 of the European Synchrotron^32^ at a wavelength of λ = 0.3998 Å. At this energy the scattering angle of the 111 reflection was *2θ* = 9.9°. The multi-analyser crystal detector at ID22 provides a high angular resolution of 0.003°, which is essential for the strain measurements. An avalanche photo diode (APD) detector was used, which possesses the sensitivity and dynamic range necessary to measure the small intensity changes associated with the resonance effects.

The X-ray wavelength was chosen according to balance between the anomalous scattering signal (imaginary parts of the atomic scattering factors, 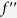) and X-ray absorption (linear attenuation coefficient, *μ*). Using the X-ray wavelength λ = 0.3998 Å means the Friedel’s pair contrast of {111} reflection was ~1% (Figs 2b and 4b). The linear attenuation coefficient of 40 cm^−1^ results in 0.73% beam transmission through the sample. Shifting the X-ray wavelength to e.g. λ = 0.33 Å (near the K-edge of Ba atom) could increase the Friedel’s pair contrast of {111} Bragg reflection up to ~4%. However this would also increase the linear attenuation coefficient up to 110 cm^−1^, resulting in only 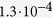 % of transmitted intensity through the sample.

The sample was fixed in a bespoke holder in which the electric field was applied perpendicular to the X-ray beam in the scattering plane and at an angle θ = 4.95° to the scattering vector (c.f. Fig. 1a). A 50 Hz, bipolar high-voltage load applied to the sample using a combination of a function generator (Hameg, Germany) and a high voltage amplifier (Matsusada, Japan). Each cycle of applied electric field comprised a pair of positive pulses, followed by a pair of negative pulses (each pulse is 2 ms long) with the maximum amplitude of 27 kV/cm. Such a waveform was designed to combine the probes of the ferroelectric and dielectric behaviours by reversing the polarization with the first pulse and probing the purely dielectric response with the second pulse. The electric current was continuously monitored via the 1 kΩ active probe and later integrated to obtain the polarization (*P-E* loops in the Figs 2d and 5a).

The time-resolved X-ray diffraction data were acquired using a bespoke stroboscopic data acquisition system operating on the principle of a multi-channel analyser, and providing an ideal platform for the investigation of repetitive processes down to the nanosecond time scale^19^,^21^,^33^. Similar stroboscopic approaches have previously been applied to the determination of small (~10^−4^ Å) electric field induced bond distortions^18^,^19^,^20^, the determination of piezoelectric coefficients^34^, the study of ferroelectric ceramics^11^,^12^,^35^ and single crystals^22^. In the configuration used here, the system used 10000 time channels of width 2 μs, later binned by a factor of 20 to improve the counting statistics at a temporal resolution (i.e. bin size) of 40 μs. The diffraction profiles were measured in a step mode (0.0005°/step, 40 sec per step). The scans were repeated 19 times, until satisfactory data statistics were collected. The entire data collection lasted for approximately 18 hours. The profiles, corresponding to each of these time-channels, 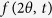 were analysed to integrate the intensities and extract the positions of the profiles mass centres according to


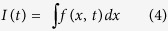



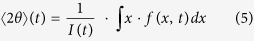


All the data analysis was performed using MATLAB (MathWorks) software.

## Additional Information

**How to cite this article**: Gorfman, S. *et al.* Simultaneous resonant x-ray diffraction measurement of polarization inversion and lattice strain in polycrystalline ferroelectrics. *Sci. Rep.* **6**, 20829; doi: 10.1038/srep20829 (2016).
